# The ‘Digital Twin’ to enable the vision of precision cardiology

**DOI:** 10.1093/eurheartj/ehaa159

**Published:** 2020-03-04

**Authors:** Jorge Corral-Acero, Francesca Margara, Maciej Marciniak, Cristobal Rodero, Filip Loncaric, Yingjing Feng, Andrew Gilbert, Joao F Fernandes, Hassaan A Bukhari, Ali Wajdan, Manuel Villegas Martinez, Mariana Sousa Santos, Mehrdad Shamohammdi, Hongxing Luo, Philip Westphal, Paul Leeson, Paolo DiAchille, Viatcheslav Gurev, Manuel Mayr, Liesbet Geris, Pras Pathmanathan, Tina Morrison, Richard Cornelussen, Frits Prinzen, Tammo Delhaas, Ada Doltra, Marta Sitges, Edward J Vigmond, Ernesto Zacur, Vicente Grau, Blanca Rodriguez, Espen W Remme, Steven Niederer, Peter Mortier, Kristin McLeod, Mark Potse, Esther Pueyo, Alfonso Bueno-Orovio, Pablo Lamata

**Affiliations:** Department of Engineering Science, University of Oxford, Oxford, UK; Department of Computer Science, British Heart Foundation Centre of Research Excellence, University of Oxford, Oxford, UK; Department of Biomedical Engineering, Division of Imaging Sciences and Biomedical Engineering, King’s College London, London, UK; Department of Biomedical Engineering, Division of Imaging Sciences and Biomedical Engineering, King’s College London, London, UK; Institut Clínic Cardiovascular, Hospital Clínic, Universitat de Barcelona, Institut d’Investigacions Biomèdiques August Pi i Sunyer (IDIBAPS), Barcelona, Spain; IHU Liryc, Electrophysiology and Heart Modeling Institute, fondation Bordeaux Université, Pessac-Bordeaux F-33600, France; IMB, UMR 5251, University of Bordeaux, Talence F-33400, France; GE Vingmed Ultrasound AS, Horton, Norway; Department of Biomedical Engineering, Division of Imaging Sciences and Biomedical Engineering, King’s College London, London, UK; IMB, UMR 5251, University of Bordeaux, Talence F-33400, France; Aragón Institute of Engineering Research, Universidad de Zaragoza, IIS Aragón, Zaragoza, Spain; The Intervention Centre, Oslo University Hospital, Rikshospitalet, Oslo, Norway; The Intervention Centre, Oslo University Hospital, Rikshospitalet, Oslo, Norway; FEops NV, Ghent, Belgium; CARIM School for Cardiovascular Diseases, Maastricht University, Maastricht, The Netherlands; CARIM School for Cardiovascular Diseases, Maastricht University, Maastricht, The Netherlands; Medtronic PLC, Bakken Research Center, Maastricht, the Netherlands; Radcliffe Department of Medicine, Division of Cardiovascular Medicine, Oxford Cardiovascular Clinical Research Facility, John Radcliffe Hospital, University of Oxford, Oxford, UK; Healthcare and Life Sciences Research, IBM T.J. Watson Research Center, Yorktown Heights, NY, USA; Healthcare and Life Sciences Research, IBM T.J. Watson Research Center, Yorktown Heights, NY, USA; King’s British Heart Foundation Centre, King’s College London, London, UK; Virtual Physiological Human Institute, Leuven, Belgium; Center for Devices and Radiological Health, U.S. Food and Drug Administration, Silver Spring, MD, USA; Center for Devices and Radiological Health, U.S. Food and Drug Administration, Silver Spring, MD, USA; Medtronic PLC, Bakken Research Center, Maastricht, the Netherlands; CARIM School for Cardiovascular Diseases, Maastricht University, Maastricht, The Netherlands; CARIM School for Cardiovascular Diseases, Maastricht University, Maastricht, The Netherlands; Institut Clínic Cardiovascular, Hospital Clínic, Universitat de Barcelona, Institut d’Investigacions Biomèdiques August Pi i Sunyer (IDIBAPS), Barcelona, Spain; Institut Clínic Cardiovascular, Hospital Clínic, Universitat de Barcelona, Institut d’Investigacions Biomèdiques August Pi i Sunyer (IDIBAPS), Barcelona, Spain; CIBERCV, Instituto de Salud Carlos III, (CB16/11/00354), CERCA Programme/Generalitat de, Catalunya, Spain; IHU Liryc, Electrophysiology and Heart Modeling Institute, fondation Bordeaux Université, Pessac-Bordeaux F-33600, France; IMB, UMR 5251, University of Bordeaux, Talence F-33400, France; Department of Engineering Science, University of Oxford, Oxford, UK; Department of Engineering Science, University of Oxford, Oxford, UK; Department of Computer Science, British Heart Foundation Centre of Research Excellence, University of Oxford, Oxford, UK; The Intervention Centre, Oslo University Hospital, Rikshospitalet, Oslo, Norway; Department of Biomedical Engineering, Division of Imaging Sciences and Biomedical Engineering, King’s College London, London, UK; FEops NV, Ghent, Belgium; GE Vingmed Ultrasound AS, Horton, Norway; IHU Liryc, Electrophysiology and Heart Modeling Institute, fondation Bordeaux Université, Pessac-Bordeaux F-33600, France; IMB, UMR 5251, University of Bordeaux, Talence F-33400, France; Inria Bordeaux Sud-Ouest, CARMEN team, Talence F-33400, France; Aragón Institute of Engineering Research, Universidad de Zaragoza, IIS Aragón, Zaragoza, Spain; CIBER in Bioengineering, Biomaterials and Nanomedicine (CIBER‐BBN), Madrid, Spain; Department of Computer Science, British Heart Foundation Centre of Research Excellence, University of Oxford, Oxford, UK; Department of Biomedical Engineering, Division of Imaging Sciences and Biomedical Engineering, King’s College London, London, UK

**Keywords:** Precision medicine, Digital twin, Computational modelling, Artificial intelligence

## Abstract

Providing therapies tailored to each patient is the vision of precision medicine, enabled by the increasing ability to capture extensive data about individual patients. In this position paper, we argue that the second enabling pillar towards this vision is the increasing power of computers and algorithms to learn, reason, and build the ‘digital twin’ of a patient. Computational models are boosting the capacity to draw diagnosis and prognosis, and future treatments will be tailored not only to current health status and data, but also to an accurate projection of the pathways to restore health by model predictions. The early steps of the digital twin in the area of cardiovascular medicine are reviewed in this article, together with a discussion of the challenges and opportunities ahead. We emphasize the synergies between mechanistic and statistical models in accelerating cardiovascular research and enabling the vision of precision medicine.

**Figure:**
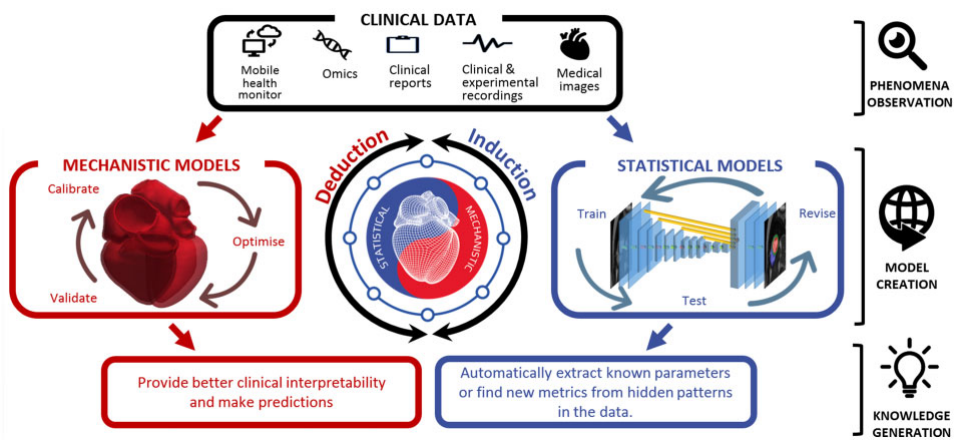

## Introduction

Providing therapies that are tailored to each patient, and that maximize the efficacy and efficiency of our healthcare system, is the broad goal of precision medicine. The main shift from current clinical practice is to take inter-individual variability into greater account. This exciting vision has been championed by the -omics revolution, i.e., the increasing ability to capture extensive data about the pathophysiology of the patient.^1^ ^,^ ^2^ This -omics approach has already delivered great achievements, especially in the management of specific cancer conditions.^3^ Nevertheless, the initial conception of precision medicine has already been criticized for being too centred in genomics and failing to address challenges of clinical management.^4^ The concept is thus gradually widening, shifting from the original gene-centric perspective to the wide spectrum of lifestyle, environment, and biology data.^5^ ^,^ ^6^

In this context, we argue that the definition of optimal therapy options requires a mechanistic understanding that links all levels from genetic and molecular traces to the pathophysiology, lifestyle and environment of the patient, and back. Precision medicine requires, not only better and more detailed data, but also the increasing ability of computers to analyse, integrate, and exploit these data, and to construct the ‘digital twin’ of a patient. In health care, the ‘digital twin’ denotes the vision of a comprehensive, virtual tool that integrates coherently and dynamically the clinical data acquired over time for an individual using mechanistic and statistical models.^7^ This borrows but expands the concept of ‘digital twin’ used in engineering industries, where *in* *silico* representations of a physical system, such as an engine or a wind farm, are used to optimize design or control processes, with a real-time connection between the physical system and the model.^8^

**Figure 1:**
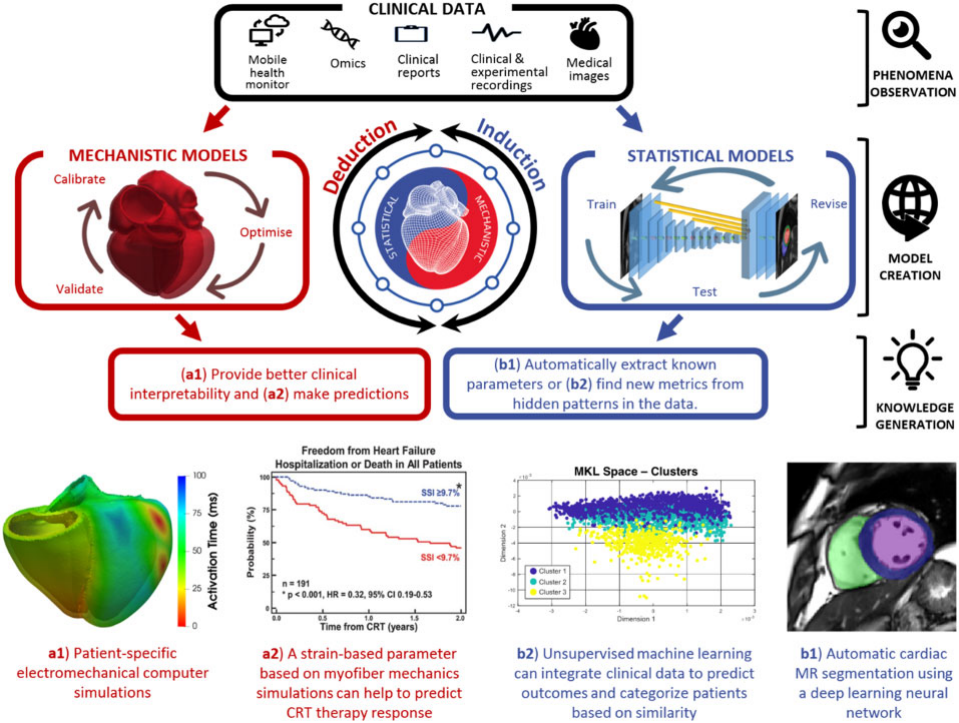
The two pillars of the digital twin, mechanistic and statistical models, illustrating its construction and four examples of use: **a1**,^10^  **a2**,^11^  **b1**,^12^  **b2**.^13^

This position paper claims that precision cardiology will be delivered in a synergetic fashion that combines induction, by using statistical models learnt from data, and deduction, through mechanistic modelling and simulation integrating multiscale knowledge and data.^9^ These are the two pillars of the digital twin (*Figure 1*). We review the state of the art of the interplay between such models that supports this vision, considering that there are already excellent independent review papers in the fields of statistical^14–16^ and mechanistic^17^ ^,^ ^18^ models for cardiovascular medicine.

Mechanistic models encapsulate our knowledge of physiology and the fundamental laws of physics and chemistry. They provide a framework to integrate and augment experimental and clinical data, enabling the identification of mechanisms and/or the prediction of outcomes, even under unseen scenarios without the need for retraining.^19^ Examples of such mechanistic models are the bidomain equations for cardiac electrophysiology^20^ or the Navier–Stokes equations for coronary blood flow.^21^ In a complementary manner, statistical models encapsulate the knowledge and relations induced from the data. They allow the extraction and optimal combination of individualized biomarkers with mathematical rules. Examples of statistical models applied to computational cardiology are random forests for assessment of heart failure severity^22^ or Gaussian processes to capture heart rate variability.^23^

**Figure 2:**
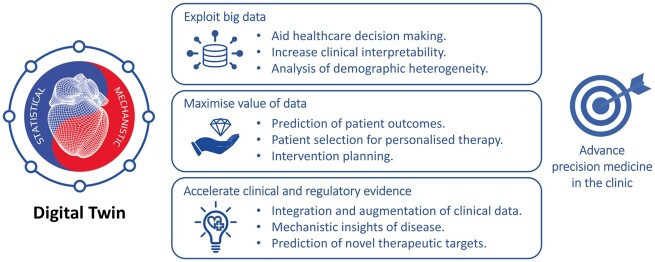
Conceptual summary of the main benefits of digital twin technologies.

There are clinical needs that can be solved with a single modelling approach. But both mechanistic and statistical models have limitations that can be addressed by combining them. Mechanistic models are constrained by their premises (assumptions and principles), while statistical models are constrained by the observations available (the amount and diversity of data). A mechanistic model may be a good choice when a good understanding of the system is available. A statistical model, on the other hand, can serve to find predictive relations even when the underlying mechanisms are poorly understood or are too complex to be modelled mechanistically. The rest of the article describes the synergies between mechanistic and statistic models (see *Figure 2* for an overview), motivated by actual clinical problems and needs, with specific representative components of the digital twin. Supplementary material online reviews the model synergies for exploiting and integrating clinical data.

## Mechanistic and statistical model synergy for improving clinical decisions

**Figure 3:**
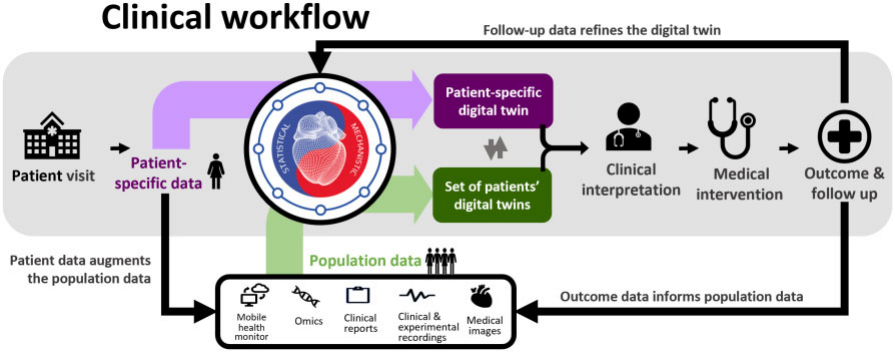
Envisioned clinical workflow using the fully developed digital twin concept. Population data, collected from preceding patients and study cohorts, are used to create and validate statistical and mechanistic models, as well as to create a population-based digital twin (green). Novel patient data are analysed with the help of the existing models and integrated to form the patient’s digital twin (purple). The comparison and interaction between digital twins give valuable insight (phenotyping, risk assessment, prediction of disease development…) that is clinically interpreted and combined with traditional data to aid in the process of clinical decision-making. The digital twin develops in line with the patient’s condition—adjusting and improving in accordance with the follow-up data. Resulting outcomes are supplemented to shape population data and refine the follow-up data.

Technical, ethical, and financial constraints limit the data acquisition needed to assist clinical decision-making.^14^ ^,^ ^15^ Synergy between mechanistic and statistical models has shown value in aiding diagnosis, treatment, and prognosis evaluation. A fully developed digital twin will combine population and individual representations to optimally inform clinical decisions (*Figure 3*).

### Model synergy in aiding diagnosis

Models can pinpoint the most valuable piece of diagnostic data. An example is the simulation study that revealed that fibrosis and other pulmonary vein properties may better characterize susceptibility to atrial fibrillation.^24^ Models can also reliably infer biomarkers that cannot be directly measured or that require invasive procedures. For instance, the combination of cardiovascular imaging and computational fluid dynamics enables non-invasive characterizations of flow fields and the calculation of diagnostic metrics in the domains of coronary artery disease, aortic aneurysm, aortic dissection, valve prostheses, and stent design.^25–29^

The key to guide diagnosis is the personalization of a mechanistic model to the actual health status of the patient as captured in available clinical data. In this personalization process, statistical models enable robust and reproducible analysis of clinical data and infer missing parameters. An example of this synergy is the assessment of left ventricular myocardial stiffness and decaying diastolic active tension by fitting mechanical models to pressure data and images during diastole.^30^ ^,^ ^31^ Another example is the non-invasive computation of pressure drops in flow obstructions,^32^ ^,^ ^33^ such as aortic stenosis or aortic coarctation, which has been proven more accurate than methods recommended in clinical guidelines.^34^ Models have also been used to derive fractional flow reserve from computed tomography (CT) to non-invasively identify ischaemia in patients with suspected coronary artery disease, avoiding invasive catheterized procedures.^29^ ^,^ ^35–37^

Some diagnostic medical devices based on personalized mechanistic models have already reached their industrial translation and clinical adoption. HeartFlow FFR_CT_ Analysis (HeartFlow, USA) and CardioInsight (Medtronic, USA) use patient-specific mechanistic models to non-invasively calculate clinically relevant diagnostic indexes and have received clearance from the USA Food and Drug Administration (FDA).^38^ HeartFlow predicts fractional flow reserve by means of a personalized 3D model of blood flow in the coronary arteries.^36^ In the CardioInsight mapping system, the electrical activity on the heart surface is recovered from body surface potentials using a personalized model of the patient’s heart and torso.^39^

### Model synergy in guiding treatments

A digital twin may indicate whether a medical device or pharmaceutical treatment is appropriate for a patient by simulating device response or dosage effects before a specific therapy is selected.

The benefits of cardiac resynchronization therapy (CRT) have been demonstrated in patients with prolonged QRS duration. However, uncertainty remains in patients with more intermediate electrocardiogram (ECG) criteria.^40^ To guide decision-making in this ‘grey zone’, approaches using mechanistic modelling have investigated the role of different aetiologies of mechanical discoordination in CRT response.^10^ For example, a novel radial strain-based metric was defined based on simulations of the human heart and circulation to differentiate patterns of mechanical discoordination, suggesting that the response to CRT could be predicted from the presence of non-electrical substrates.^11^ Statistical methods were used to verify these findings in a clinical cohort, and the novel index remained useful in predicting response in the clinical ‘grey zone’, creating the opportunity to improve patient selection in the group with intermediate ECG.

Another example is the improvements in ablation guidance of infarct-related ventricular tachycardia, where the accurate identification of patient-specific optimal targets is provided before the clinical procedure.^41^ Mechanistic models can propose novel electro-anatomical mapping indices to locate critical sites of re-entry formation in scar-related arrhythmias, aid acquisition and quantitative interpretation of electrophysiological data, and optimize future clinical use.^42^

The industrial translation and clinical adoption of models for guiding treatment are exemplified by the optimal planning of valve prosthesis with the HEARTguide™ platform (FEops nv, Belgium), or by the platform to guide ventricular tachycardia ablations (inHeart, France).

### Model synergy in evaluating prognosis

While statistical modelling allows for categorizing patients based on the probability of various outcomes, mechanistic modelling provides more insights to support or reject the categorization.

**Figure 4:**
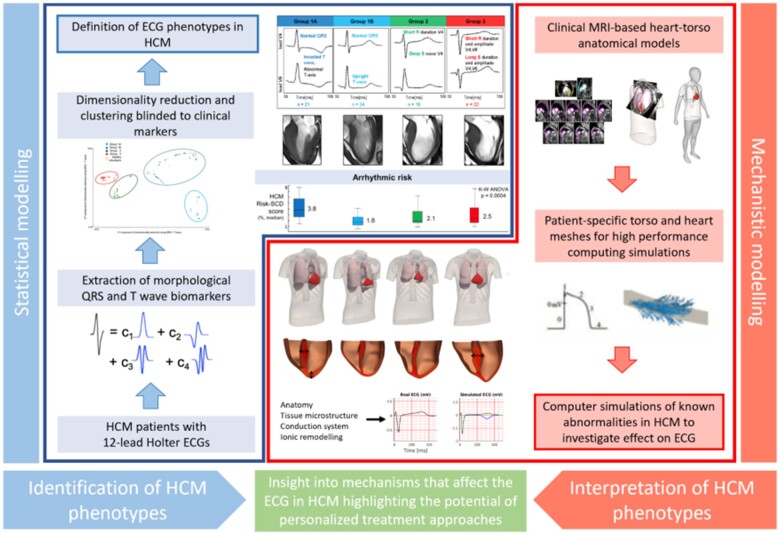
Synergy between mechanistic and statistical models in the definition of electrocardiogram (ECG) biomarkers for the management of hypertrophic cardiomyopathy.^47^ ^,^ ^48^

For example, model synergies represent an exciting approach to interpret structure–function relationships and improve risk prediction in inherited disease conditions, such as hypertrophic cardiomyopathy (HCM). Relationships among specific ECG changes, ventricle morphologies, and sudden cardiac death have been inferred from observations.^43^ ^,^ ^44^ However, the complex process of translating underlying heterogeneous substrates in HCM to ECG findings is still poorly understood, and there exists a ‘grey zone’ of clinical decision-making in the low-risk patient subgroups, specifically when deciding on restriction of involvement in professional sports.^45^ In this context, by using methods of statistical inference and mathematical modelling (see *Figure 4*), HCM patients were categorized into phenogroups based on ECG biomarkers extracted from 24-h ECG recordings,^48^ and the aetiology of each ECG phenogroup linked with different underlying substrates, suggesting ion-channel and conduction system abnormalities.^46^ ^,^ ^47^ The results directly highlighted the potential of personalized anti-arrhythmic approaches in the treatment of HCM patients, and addressed the low-risk patients, showing that a normal ECG might indeed be the discriminatory factor signalling minimal ionic remodelling, fibrosis, disarray, and ischaemia in these ‘grey zone’ patients.

Models have also been used in the prediction of arrhythmic events in post-myocardial infarction, outperforming existing clinical metrics including ejection fraction.^49^ When the amount of data is not sufficient to inform state-of-the-art machine learning methods, statistical methods can still prove useful. An example is the use of principal component analysis to account for right ventricular motion in predicting survival in pulmonary hypertension,^50^ or to identify signatures of anatomical remodelling that predict a patient’s prognosis following CRT implantation.^51^

While statistical models allow predictions, mechanistic models provide the underlying explanations. Understanding the actual meaning of the selected features improves the plausibility of findings and increases their credibility. For both approaches, quantifying uncertainty of prediction can help identify cases that may require further review, while building trust in cases where models are shown to be robust.^52^ ^,^ ^53^

## Mechanistic and statistical model synergy to accelerate evidence generation

While digital twin technologies in cardiology show promising research results, only a small number of models have reached clinical translation. The difficulties encountered include the need to increase validation, lack of clinical interpretability, and potentially obscure model failures.^54^ Therefore, solid evidence for the generalization of preliminary findings and efficient testing strategies are needed. Even when these barriers are overcome, rigid assessment of algorithmic performance and quality control from regulatory bodies can slow down the adoption. In this context, model synergy can be used to accelerate the integration of novel technologies into clinical practice by increasing clinical interpretability, validating generality of findings, and accelerating regulatory decision-making.

### Model validation towards generality of findings

The goal after validating an initial concept is to extend it to a more general patient cohort, with less controlled characteristics. The problem of sampling bias, based on both intrinsic (physiological) and extrinsic (environmental) demographic heterogeneity of the population, becomes relevant when implementing solutions for broader patient cohorts.^55^ ^,^ ^56^ Consequently, models (as clinical guidelines) may need recalibrations when used on populations from different countries or ethnicities, or even from different centres in the same country. In recent years, only 6% of artificial intelligence algorithms had external evaluation performed (note this is beyond the minimum requirement of using the learning, validation, and testing partitions of the data), and none adopted the three design criteria of a robust validation: diagnostic cohort design, the inclusion of multiple institutions, and prospective data collection.^57^ The quality of datasets also needs to be thoroughly validated to avoid possible biases before the models developed from them can be integrated in clinical decision-making.^58^

To address this issue, an increasing number of institutions are creating initiatives for data-sharing platforms, aiming at reusing existing datasets and verifying published research works.^59^ Governments, regulatory agencies, and philanthropic funders are promoting the open science culture, enforcing publishing patient-level data by means of compliance to product launching, funding application, and journal publishing.^60^

Another approach to improve the generality of data is the generation of synthetic cases of a representative wider population. The core idea is to expand the average mechanistic model to obtain populations of models, all of them parameterized within the range of physiological variability obtained by experimental protocols.^61^ ^,^ ^62^ Such an approach, which allows investigating many more scenarios than possible experimental acquisitions, is not only able to evaluate the impact of physiological variability but to explain the mechanisms underpinning inter-individual variability in therapy response (e.g. adverse drug reactions), and to identify sub-populations at higher risk.^63^ ^,^ ^64^ Statistical shape modelling techniques can represent inter-patient anatomical variability for a cohort, and be used in combination of mechanistic models for clinical decision support systems.^65^

As in traditional scientific research, mechanistic and statistical models are complementary tools to verify the findings derived from one another. Finding a mechanistic explanation of an inductive inference from statistical models increases its plausibility, such as the redistribution of work in the left bundle branch block to explain the remodelling pattern that predicts response to CRT.^51^ Equivalently, data computed from mechanistic models need to be scrutinized quantitatively as it was done in the comparison of clinical and simulation groups to validate a model for acute normovolaemic haemodilution.^66^

An important final remark is that randomized control trials will always be needed to establish evidence that can never be obtained from large observational databases.^67^

### Models as critical tools for accelerating regulatory decision-making

Clinical decisions are built on evidence from bench to bedside. Regulatory decisions, on the contrary, are often based on heterogeneous, limited, or completely absent human data, as in the case of approval for first-in-human clinical trials. In this regard, the results of computational models can now be accepted for some regulatory submissions.^68^ ^,^ ^69^ Digital evidence obtained using computer simulations can be used for safety of therapy prior to first-in-human use, or under scenarios not ethically possible in human.^38^ Computational models have an increasingly important role in the overall product life cycle management, proving useful in the processes of design optimization for development and testing, supplemental non-clinical testing, and post-market design changes and failure assessment.^27^

The development process for medical devices involves manufacturing and testing samples under a wide range of scenarios, which is often time-consuming and financially overwhelming. Moreover, pre-clinical testing conditions are often very simplified with respect to the actual patient environment. Statistical and mechanistic models synergistically offer to streamline this process, where statistical models can be used to collect a representative virtual patient cohort, and mechanistic models can then be used to simulate the device behaviour under defined scenarios. In this way, new devices can be tested in a representative virtual patient population, thereby decreasing the risk before moving to an actual clinical trial. An example is HEARTguide™ (FEops nv, Belgium), where device–patient interactions after transcatheter aortic valve implantation can be predicted.^25^

The augmentation of clinical trial design with virtual patients is also an evolving idea.^70–72^ This would overcome limitations of current empirical trials, where patients burdened with comorbidities or complex treatment regimens are often excluded from the trials, and enrolled individuals are handled under reductionist approaches, assuming they share a common phenotype. Such approaches often fail to capture differences in response to treatment.^70^ Alternatively, computational evidence can inform collection of novel evidence from clinical trials,^13^ ^,^ ^38^ where models can improve patient selection by derived biomarkers and predictions. This offers an opportunity to answer questions traditionally restricted by financial or ethical considerations, and to investigate therapy efficacy in more clinically relevant cases. Computational modelling can also facilitate safe methods to explore treatment effects in sub-populations clinically more complex to address, such as patients with rare diseases or paediatric cohorts, and therefore may allow for insights not possible in the current clinical trial practice.

One of the first examples in which digital evidence (i.e. an *in* *silico* trial) replaced any additional clinical evidence was in the approval of the Advisa MRI SureScan pacemaker (Medtronic, Inc.).^73^ Another powerful example is a computer simulator of type 1 diabetes mellitus,^74^ which was accepted by the FDA as a substitute to animal trials for the pre-clinical testing of control strategies in artificial pancreas studies. Later, an investigational device exemption (i.e. the approval needed to initiate a clinical study), issued solely on the basis of modelling testing, was granted by the FDA for a closed-loop control clinical trial of the safety and effectiveness of the proposed artificial pancreas algorithm.

In the context of drug safety and efficacy assessment, an unmet need is filling the gaps between animal translation or *in* *vitro* preparations and prediction of the human response. Mechanistic models may assist in scaling observations into humans.^75^ This is, for example, the goal of the CIPA initiative,^69^ sponsored by the FDA among others, aiming at facilitating the adoption of a new paradigm for assessment of potential risk of clinical Torsades de Pointes, where mechanistic models of human electrophysiology will play a crucial role. This is reinforced by a recent study in which human *in silico* trials outperformed animal models in predicting clinical pro-arrhythmic cardiotoxicity, so they might be soon integrated into existing drug safety assessment pipelines.^63^

Finally, after a product is launched, mechanistic models can be still used for post-market re-evaluation and failure assessment in order to identify any potential underlying problems. This creates a valuable opportunity for simulations to evaluate any design changes planned for next-generation productions, ultimately closing the product life cycle loop, and demonstrating the ubiquitous presence and utility of statistical and mechanistic models in the future of medical product regulation.

## Discussion

The digital twin, i.e., the dynamic integration and augmentation of patient data using mechanistic and statistical models, is the actual pathway towards the vision of precision medicine. Simple and fragmented components of the digital twin are already used in clinical practice: a decision tree in a clinical guideline encapsulates the best-documented evidence that is based in statistical and mechanistic insights. The digital twin will gradually include tailored computer-enabled decision points, and create the transition from healthcare systems founded on describing disease to healthcare systems focused on predicting response, and thus shifting treatment selection from being based on the state of the patient today to optimizing the state of the patient tomorrow.

### Envisioned impact and timeline

**Figure 5:**
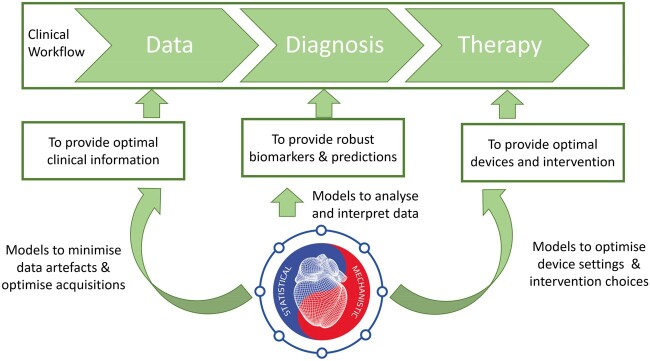
The vision of a personalized *in silico* cardiology, where the digital twin informs all the stages through the clinical workflow. Models are used (i) to optimize data acquisition and the information extracted from it, (ii) to evaluate current health status and inform diagnosis and risk stratification, and (iii) to optimize clinical devices and drug selection to deliver a personalized therapy.

The digital twin provides a pathway to map current patient observations into a predictive framework, combining inductive and deductive reasoning. Early components of the digital twin are already making a clinical impact. In a generic clinical workflow divided in the stages of data acquisition, diagnosis, and therapy planning, computational models can provide value in the three stages, see *Figure 5*. To improve data acquisition techniques, there are already statistical models to automate the image analysis tasks.^16^ To provide better diagnosis, a virtual fractional flow reserve can replace an invasive catheter,^29^ ^,^ ^37^ or the body surface recordings can be mapped to the surface of the heart.^39^ With regards to therapy planning, a virtual deployment of the valve replacement^25^ ^,^ ^76^ or a roadmap to guide ablation procedures^77^ ^,^ ^78^ represents existing techniques (statistical and mechanistic) that have been implemented into the clinical workflow. These solutions have thus met regulatory approval, where they are referred to as ‘software as a medical device’, and where guidelines from the International Medical Device Regulators Forum are accepted by the EU and the USA.

**Take home figure:**
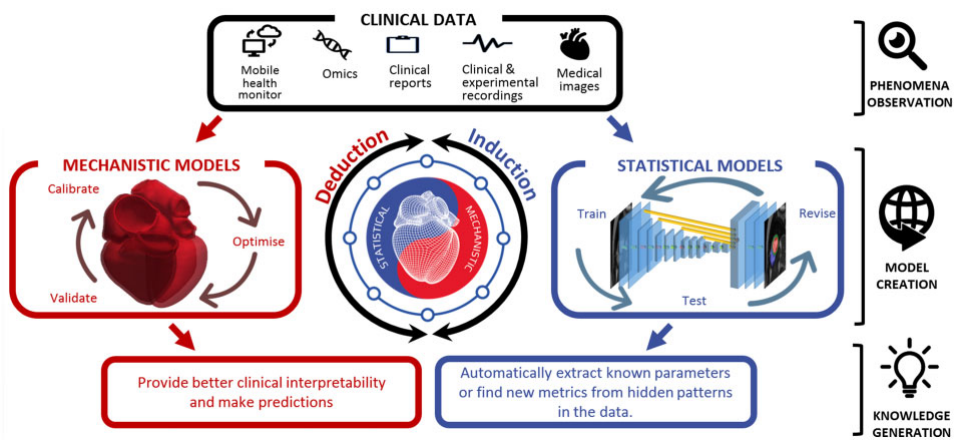
The cardiovascular digital twin that will deliver the vision of precision medicine by the synergetic combination of computer-enhanced induction (using statistical models learnt from data) and deduction (mechanistic modelling and simulation integrating multi-scale knowledge).

A digital twin will follow the life journey of each person and harness both data collected by wearable sensors and lifestyle information that patients may register, shifting the clinical approach towards preventive healthcare. A notable challenge is the integration of these data with healthcare organizations, where security and confidentiality of the sensitive information remain paramount.

The currently still fragmented and incipient concept of the digital twin will be gradually crystallized and adopted during the next 5–10 years. The holistic integration of a Digital Twin is the aspiration that will be reached through two complementary and synergetic pathways: the first is the refinement of key decision points in the management of cardiac disease, driven by personalized mechanistic models that are informed by key pieces of patient’s data; and the second is the disease-centred optimization of the patient’s lifetime journey through the healthcare system, driven by statistical models being informed by the electronic health record of a large population.

On the actual implementation of the digital twin, we envision that the evolution will be towards a gradually better inter-operability of current health information systems, leading to a distributed location of the information. Digital twin users will mainly be citizens and physicians, with different interfaces that retrieve the relevant data and trigger the analysis capabilities hosted in the local device or remote cloud resources. The analysis may also require specialized skills that may be delivered by industry, or even by *computational cardiologists* inside healthcare organizations.

### Organizational and societal challenges ahead

Access to data is the main challenge in both the development and the clinical translation of the digital twin, caused by infrastructural, regulatory, and societal reasons. Information systems and electronic health records are fragmented, highly heterogeneous and difficult to inter-operate. Information is often contained in unstructured format, and its extraction requires either manual work or further research efforts of automation through natural language processing technologies.^79^ Simulations may also require specialized skills and supercomputers. In this context, provision of digital twin technologies may be enabled by cloud infrastructures (e.g. HeartFlow FFR_CT_ Analysis).

Consent and confidentiality are key ingredients to address the societal concerns when handling the personal data needed to develop and validate digital twin technologies. The EU General Data Protection Regulation (GDPR) has imposed new legal requirements, such as the right to withdraw consent and the right to be forgotten, causing controversy about the cost and feasibility of its enforcement.^80^ Any digital twin solution that holds enough information to identify a patient needs to carefully watch these requirements, that also apply to retrospective data and safety backups.

### Potential professional, cultural, and ethical issues

As more clinical tasks are performed by models, the fear of replacement of physicians by machines may arise. In some scenarios, machines may match or even outperform physicians.^81^ In other scenarios, human experts, by not practising on the easy problems solved by the machine, may lose the skills that may still be needed when dealing with difficult cases.

The second professional barrier is the mistrust that originates from a ‘black box’, where predictions derived by algorithms are not matched with a plausible explanation. Generation of evidence is one clear way to generate trust. Another solution is to use methods to illustrate the logic inside the box, including clustering and association techniques,^82^ which may help to identify the causes and mechanisms.

From the patient’s perspective, personalization creates the opportunity of more involvement in healthcare decisions. Patients will be empowered to better manage their disease using the digital twin to gain information about their current and predicted state, and potentially to adopt optimized lifestyle suggestions. A well-informed patient shall have more efficient discussions with physicians, and consent and decide faster on diagnostic or treatment procedures.

Finally, on the ethical side, there is a risk of models to create or exacerbate existing racial or societal biases in healthcare systems: if a group is misrepresented in the data used to train models, that group may receive a sub-optimal treatment.^83^

### Recommendations

The pathway to accelerate the clinical impact with digital twin technologies is to generate trust among researchers, clinicians, and society.

Research communities shall avoid inflating expectations. Claims about generality and potential impact should be based on rigorous methodology, with external cohorts to demonstrate the validity of inferences, and with the quantification of the uncertainty of predictions.^84^ Any model is a simplified representation of the reality, with a limited scope and dependence on assumptions made. The opportunity is an adequate handling of these limitations, with models able to identify data inconsistencies, and with data used to constrain and verify the model assumptions.^85^

As an emerging field, the digital twin needs guidelines, gold-standards, and benchmark tests.^86^ ^,^ ^87^ Scientific organizations and regulatory bodies have released guidelines that can be used to establish the level of rigour needed for computational modelling.^27^ Such guidelines and standards are useful tools as they allow regulators to judge computational evidence and industry to understand regulatory requirements for computational models, leveraging a substantial part of the risk and uncertainty associated to the development of these new technologies. They can even increase and facilitate their translational impact, as the quality and robustness of the models and their reporting will increase by adhering to such guidance during model development. Further effort is needed to widen the scope of these first multi-stakeholder consensuses involving industry, academia, and regulators. Current initiatives that develop visions, technologies, or infrastructure relevant to the ‘digital twin’ community are Elixir (https://elixir-europe.org/), FAIRDOM (https://fair-dom.org/), and EOSC (https://ec.europa.eu/research/openscience/index.cfm? pg=open-science-cloud).

The education of citizens, care providers, physicians, and researchers in the uses and possibilities of digital twin technologies is key for its adoption and acceptance. University education systems should also allow for the exchange of knowledge at the earliest stages of the career: medical students should have some computational training, just as engineers in biomedical industry should be trained in cardiology during their studies.^88^ And postgraduate training programmes should bridge remaining cultural and language gaps between disciplines, such as our Personalised In-silico Cardiology EU funded Innovative Training Network (https://picnet.eu).

## Conclusion

Precision cardiology will be delivered, not only by data, but also by the inductive and deductive reasoning built in the digital twin of each patient. Treatment and prevention of cardiovascular disease will be based on accurate predictions of both the underlying causes of disease and the pathways to sustain or restore health. These predictions will be provided and validated by the synergistic interplay between mechanistic and statistical models. The early steps towards this vision have been taken, and the next ones depend on the coordinated drive from scientific, clinical, industrial, and regulatory stakeholders in order to build the evidence and tackle the organizational and societal challenges ahead.

## Supplementary material

Supplementary material is available at *European Heart Journal* online.

**Conflict of interest:** none declared.

## Funding

This work was supported by the EU’s Horizon 2020 Marie Sklodowska-Curie ITN Projects (g.a. 764738 and 766082), the EU’s Horizon 2020 research and innovation programme (g.a. 675451 and 823712), the Wellcome/EPSRC Centre for Medical Engineering (WT 203148/Z/16/Z), the National Research Agency (ANR) (g.a. ANR-10-IAHU-04), the NC3RS (NC/P001076/1) and the British Heart Foundation (RE/13/2/30182, RE/13/1/30181, TG/17/3/33406, PG/16/75/32383, FS/17/22/32644, CH/16/3/21406, RG/16/14/32397). E.Pueyo holds an ERC Starting Grant (g.a. 638284). B. Rodriguez and P.Lamata hold Wellcome Trust Senior Research Fellowships (214290/Z/18/Z, 209450/Z/17/Z).

## Supplementary Material

ehaa159_Supplementary_Data

## References

[1] Antman EM, Loscalzo J. Precision medicine in cardiology. Nat Rev Cardiol 2016;13:591–602. 27356875 10.1038/nrcardio.2016.101

[2] Trayanova N. From genetics to smart watches: developments in precision cardiology. Nat Rev Cardiol 2019;16:72–73. 30568275 10.1038/s41569-018-0149-y PMC6550458

[3] Collins FS, Varmus H. A new initiative on precision medicine. N Engl J Med 2015;372:793–795. 25635347 10.1056/NEJMp1500523 PMC5101938

[4] Joyner MJ, Paneth N. Promises, promises, and precision medicine. J Clin Invest 2019;129:946–948. 30688663 10.1172/JCI126119 PMC6391102

[5] Khoury MJ. Precision medicine vs preventive medicine. JAMA 2019;321:406. 10.1001/jama.2018.18636 30694310

[6] Noble D. Evolution beyond neo-Darwinism: a new conceptual framework. J Exp Biol 2015;218:7–13. 25568446 10.1242/jeb.106310

[7] Alber M, Buganza Tepole A, Cannon WR, De S, Dura-Bernal S, Garikipati K, Karniadakis G, Lytton WW, Perdikaris P, Petzold L, Kuhl E. Integrating machine learning and multiscale modeling—perspectives, challenges, and opportunities in the biological, biomedical, and behavioral sciences. NPJ Digit Med 2019;2:115. 31799423 10.1038/s41746-019-0193-y PMC6877584

[8] Tao F, Cheng J, Qi Q, Zhang M, Zhang H, Sui F. Digital twin-driven product design, manufacturing and service with big data. Int J Adv Manuf Technol 2018;94:3563–3576.

[9] Lamata P. Teaching cardiovascular medicine to machines. Cardiovasc Res 2018;114:e62–e64. 29850780 10.1093/cvr/cvy127 PMC6014504

[10] Niederer SA, Plank G, Chinchapatnam P, Ginks M, Lamata P, Rhode KS, Rinaldi CA, Razavi R, Smith NP. Length-dependent tension in the failing heart and the efficacy of cardiac resynchronization therapy. Cardiovasc Res 2011;89:336–343. 20952413 10.1093/cvr/cvq318

[11] Lumens J, Tayal B, Walmsley J, Delgado-Montero A, Huntjens PR, Schwartzman D, Althouse AD, Delhaas T, Prinzen FW, Gorcsan J. Differentiating electromechanical from non-electrical substrates of mechanical discoordination to identify responders to cardiac resynchronization therapy. Circ Cardiovasc Imaging 2015;8:e003744. 26338877 10.1161/CIRCIMAGING.115.003744

[12] Corral Acero J, Zacur E, Xu H, Ariga R, Bueno-Orovio A, Lamata P, Grau V. SMOD - Data Augmentation Based on Statistical Models of Deformation to Enhance Segmentation in 2D Cine Cardiac MRI. FIMH 2019: Functional Imaging and Modeling of the Heart - pp. 361–369. doi: 10.1007/978-3-030-21949-9_39.

[13] Cikes M, Sanchez Martinez S, Claggett B, Solomon SD, Bijnens B. Machine-learning integration of complex echocardiographic patterns and clinical parameters from cohorts and trials. Eur Heart J 2019;40: doi: 10.1093/eurheartj/ehz745.0147.

[14] Shameer K, Johnson KW, Glicksberg BS, Dudley JT, Sengupta PP. Machine learning in cardiovascular medicine: are we there yet? Heart 2018;104:1156–1164. 29352006 10.1136/heartjnl-2017-311198

[15] Rumsfeld JS, Joynt KE, Maddox TM. Big data analytics to improve cardiovascular care: promise and challenges. Nat Rev Cardiol 2016;13:350–359. 27009423 10.1038/nrcardio.2016.42

[16] Dey D, Slomka PJ, Leeson P, Comaniciu D, Shrestha S, Sengupta PP, Marwick TH. Artificial intelligence in cardiovascular imaging: JACC state-of-the-art review. J Am Coll Cardiol 2019;73:1317–1335. 30898208 10.1016/j.jacc.2018.12.054 PMC6474254

[17] Niederer SA, Lumens J, Trayanova NA. Computational models in cardiology. Nat Rev Cardiol 2019;16:100–111. 30361497 10.1038/s41569-018-0104-y PMC6556062

[18] Johnson KW, Shameer K, Glicksberg BS, Readhead B, Sengupta PP, Björkegren JLM, Kovacic JC, Dudley JT. Enabling precision cardiology through multiscale biology and systems medicine. JACC Basic Transl Sci 2017;2:311–327. 30062151 10.1016/j.jacbts.2016.11.010 PMC6034501

[19] Davies MR, Wang K, Mirams GR, Caruso A, Noble D, Walz A, Lavé T, Schuler F, Singer T, Polonchuk L. Recent developments in using mechanistic cardiac modelling for drug safety evaluation. Drug Discov Today 2016;21:924–938. 26891981 10.1016/j.drudis.2016.02.003 PMC4909717

[20] Tung L. A bi-domain model for describing ischemic myocardial D-C potentials. 1978. https://dspace.mit.edu/handle/1721.1/16177 (29 February 2020).

[21] Sherwin SJ, Formaggia L, Peiró J, Franke V. Computational modelling of 1D blood flow with variable mechanical properties and its application to the simulation of wave propagation in the human arterial system. Int J Numer Methods Fluids 2003;43:673–700.

[22] Guidi G, Pettenati MC, Miniati R, Iadanza E. Random forest for automatic assessment of heart failure severity in a telemonitoring scenario. In: *2013 35th Annual International Conference of the IEEE Engineering in Medicine and Biology Society (EMBC)*. IEEE, pp. 3230–3233. 10.1109/EMBC.2013.6610229 24110416

[23] Stegle O, Fallert SV, MacKay DJ, Brage S. Gaussian process robust regression for noisy heart rate data. IEEE Trans Biomed Eng 2008;55:2143–2151. 18713683 10.1109/TBME.2008.923118

[24] Roney CH, Bayer JD, Cochet H, Meo M, Dubois R, Jaïs P, Vigmond EJ. Variability in pulmonary vein electrophysiology and fibrosis determines arrhythmia susceptibility and dynamics. PLoS Comput Biol 2018;14:e1006166. 29795549 10.1371/journal.pcbi.1006166 PMC5997352

[25] de Jaegere P, De Santis G, Rodriguez-Olivares R, Bosmans J, Bruining N, Dezutter T, Rahhab Z, El Faquir N, Collas V, Bosmans B, Verhegghe B, Ren C, Geleinse M, Schultz C, van Mieghem N, De Beule M, Mortier P. Patient-specific computer modeling to predict aortic regurgitation after transcatheter aortic valve replacement. JACC Cardiovasc Interv 2016;9:508–512. 26965945 10.1016/j.jcin.2016.01.003

[26] Gray RA, Pathmanathan P. Patient-specific cardiovascular computational modeling: diversity of personalization and challenges. J Cardiovasc Transl Res 2018;11:80–88. 29512059 10.1007/s12265-018-9792-2 PMC5908828

[27] Morrison TM, Dreher ML, Nagaraja S, Angelone LM, Kainz W. The role of computational modeling and simulation in the total product life cycle of peripheral vascular devices. J Med Device 2017;11:024503. 29479395 10.1115/1.4035866 PMC5823268

[28] Dillon-Murphy D, Noorani A, Nordsletten D, Figueroa CA. Multi-modality image-based computational analysis of haemodynamics in aortic dissection. Biomech Model Mechanobiol 2016;15:857–876. 26416312 10.1007/s10237-015-0729-2 PMC4945697

[29] Morris PD, van de Vosse FN, Lawford PV, Hose DR, Gunn JP. “Virtual” (computed) fractional flow reserve. JACC Cardiovasc Interv 2015;8:1009–1017. 26117471 10.1016/j.jcin.2015.04.006 PMC4726733

[30] Xi J, Lamata P, Niederer S, Land S, Shi W, Zhuang X, Ourselin S, Duckett SG, Shetty AK, Rinaldi CA, Rueckert D, Razavi R, Smith NP. The estimation of patient-specific cardiac diastolic functions from clinical measurements. Med Image Anal 2013;17:133–146. 23153619 10.1016/j.media.2012.08.001 PMC6768802

[31] Wang ZJ, Wang VY, Bradley CP, Nash MP, Young AA, Cao JJ. Left ventricular diastolic myocardial stiffness and end-diastolic myofibre stress in human heart failure using personalised biomechanical analysis. J Cardiovasc Transl Res 2018;11:346–356. 29998358 10.1007/s12265-018-9816-y

[32] Krittian SBS, Lamata P, Michler C, Nordsletten DA, Bock J, Bradley CP, Pitcher A, Kilner PJ, Markl M, Smith NP. A finite-element approach to the direct computation of relative cardiovascular pressure from time-resolved MR velocity data. Med Image Anal 2012;16:1029–1037. 22626833 10.1016/j.media.2012.04.003 PMC3387378

[33] Donati F, Figueroa CA, Smith NP, Lamata P, Nordsletten DA. Non-invasive pressure difference estimation from PC-MRI using the work-energy equation. Med Image Anal 2015;26:159–172. 10.1016/j.media.2015.08.012 PMC4686008 26409245

[34] Donati F, Myerson S, Bissell MM, Smith NP, Neubauer S, Monaghan MJ, Nordsletten DA, Lamata P. Beyond Bernoulli: improving the accuracy and precision of non-invasive estimation of peak pressure drops. Circ Cardiovasc Imaging 2017;10:e005207. 28093412 10.1161/CIRCIMAGING.116.005207 PMC5265685

[35] Nørgaard BL, Leipsic J, Gaur S, Seneviratne S, Ko BS, Ito H, Jensen JM, Mauri L, De Bruyne B, Bezerra H, Osawa K, Marwan M, Naber C, Erglis A, Park S-J, Christiansen EH, Kaltoft A, Lassen JF, Bøtker HE, Achenbach S. Diagnostic performance of noninvasive fractional flow reserve derived from coronary computed tomography angiography in suspected coronary artery disease. J Am Coll Cardiol 2014;63:1145–1155. 24486266 10.1016/j.jacc.2013.11.043

[36] Min JK, Taylor CA, Achenbach S, Koo BK, Leipsic J, Nørgaard BL, Pijls NJ, De Bruyne B. Noninvasive fractional flow reserve derived from coronary CT angiography. JACC Cardiovasc Imaging 2015;8:1209–1222. 26481846 10.1016/j.jcmg.2015.08.006

[37] Rajani R, Modi B, Ntalas I, Curzen N. Non-invasive fractional flow reserve using computed tomographic angiography: where are we now and where are we going? Heart 2017;103:1216–1222. 28559426 10.1136/heartjnl-2016-311029

[38] Morrison TM, Pathmanathan P, Adwan M, Margerrison E. Advancing regulatory science with computational modeling for medical devices at the FDA’s Office of Science and Engineering Laboratories. Front Med 2018;5: doi: 10.3389/fmed.2018.00241. 10.3389/fmed.2018.00241 PMC6167449 30356350

[39] Haissaguerre M, Hocini M, Shah AJ, Derval N, Sacher F, Jais P, Dubois R. Noninvasive panoramic mapping of human atrial fibrillation mechanisms: a feasibility report noninvasive panoramic mapping of human atrial fibrillation mechanisms. Introduction. J Cardiovasc Electrophysiol 2013;24:711–717. 23373588 10.1111/jce.12075

[40] Daubert C, Behar N, Martins RP, Mabo P, Leclercq C,. Avoiding non-responders to cardiac resynchronization therapy: a practical guide. Eur Heart J 2016;38:1463–1472. 10.1093/eurheartj/ehw270 27371720

[41] Prakosa A, Arevalo HJ, Deng D, Boyle PM, Nikolov PP, Ashikaga H, Blauer JJE, Ghafoori E, Park CJ, Blake RC, Han FT, MacLeod RS, Halperin HR, Callans DJ, Ranjan R, Chrispin J, Nazarian S, Trayanova NA. Personalized virtual-heart technology for guiding the ablation of infarct-related ventricular tachycardia. Nat Biomed Eng 2018;2:732–740. 30847259 10.1038/s41551-018-0282-2 PMC6400313

[42] Hill YR, Child N, Hanson B, Wallman M, Coronel R, Plank G, Rinaldi CA, Gill J, Smith NP, Taggart P, Bishop MJ. Investigating a novel activation-repolarisation time metric to predict localised Vulnerability to reentry using computational modelling. PLoS One 2016;11: doi: 10.1371/journal.pone.0149342. 10.1371/journal.pone.0149342 PMC4775046 26934736

[43] Alfonso F, Nihoyannopoulos P, Stewart J, Dickie S, Lemery R, McKenna WJ. Clinical significance of giant negative T waves in hypertrophic cardiomyopathy. J Am Coll Cardiol 1990;15:965–971. 2312983 10.1016/0735-1097(90)90225-e

[44] Pelliccia A, Di Paolo FM, Quattrini FM, Basso C, Culasso F, Popoli G, De Luca R, Spataro A, Biffi A, Thiene G, Maron BJ. Outcomes in athletes with marked ECG repolarization abnormalities. *N Engl J Med* 2008;358:152–161. 10.1056/NEJMoa060781 18184960

[45] Pelliccia A, Corrado D, Bjørnstad HH, Panhuyzen-Goedkoop N, Urhausen A, Carre F, Anastasakis A, Vanhees L, Arbustini E, Priori S. Recommendations for participation in competitive sport and leisure-time physical activity in individuals with cardiomyopathies, myocarditis and pericarditis. Eur J Prev Cardiol 2006;13:876–885. 10.1097/01.hjr.0000238393.96975.32 17143118

[46] Passini E, Mincholé A, Coppini R, Cerbai E, Rodriguez B, Severi S, Bueno-Orovio A. Mechanisms of pro-arrhythmic abnormalities in ventricular repolarisation and anti-arrhythmic therapies in human hypertrophic cardiomyopathy. J Mol Cell Cardiol 2016;96:72–81. 26385634 10.1016/j.yjmcc.2015.09.003 PMC4915817

[47] Lyon A, Bueno-Orovio A, Zacur E, Ariga R, Grau V, Neubauer S, Watkins H, Rodriguez B, Mincholé A. Electrocardiogram phenotypes in hypertrophic cardiomyopathy caused by distinct mechanisms: apico-basal repolarization gradients vs. Purkinje-myocardial coupling abnormalities. Europace 2018;20:III102–III112. 30476051 10.1093/europace/euy226 PMC6251182

[48] Lyon A, Ariga R, Mincholé A, Mahmod M, Ormondroyd E, Laguna P, de Freitas N, Neubauer S, Watkins H, Rodriguez B. Distinct ECG phenotypes identified in hypertrophic cardiomyopathy using machine learning associate with arrhythmic risk markers. Front Physiol 2018;9:213. 29593570 10.3389/fphys.2018.00213 PMC5859357

[49] Arevalo HJ, Vadakkumpadan F, Guallar E, Jebb A, Malamas P, Wu KC, Trayanova NA. Arrhythmia risk stratification of patients after myocardial infarction using personalized heart models. Nat Commun 2016;7: doi: 10.1038/ncomms11437. 10.1038/ncomms11437 PMC4866040 27164184

[50] Dawes TJW, de Marvao A, Shi W, Fletcher T, Watson GMJ, Wharton J, Rhodes CJ, Howard LSGE, Gibbs JSR, Rueckert D, Cook SA, Wilkins MR, O’Regan DP. Machine learning of three-dimensional right ventricular motion enables outcome prediction in pulmonary hypertension: a cardiac MR imaging study. Radiology 2017;283:381–390. 28092203 10.1148/radiol.2016161315 PMC5398374

[51] Warriner DR, Jackson T, Zacur E, Sammut E, Sheridan P, Hose DR, Lawford P, Razavi R, Niederer SA, Rinaldi CA, Lamata P. An asymmetric wall-thickening pattern predicts response to cardiac resynchronization therapy. JACC Cardiovasc Imaging 2018;11:1545–1546. 29550311 10.1016/j.jcmg.2018.01.022 PMC6288240

[52] Mirams GR, Pathmanathan P, Gray RA, Challenor P, Clayton RH. Uncertainty and variability in computational and mathematical models of cardiac physiology. J Physiol 2016;594:6833–6847. 26990229 10.1113/JP271671 PMC5134370

[53] Pathmanathan P, Gray RA. Ensuring reliability of safety-critical clinical applications of computational cardiac models. Front Physiol 2013;4:358. 24376423 10.3389/fphys.2013.00358 PMC3858646

[54] Winslow RL, Trayanova N, Geman D, Miller MI. Computational medicine: translating models to clinical care. Sci Transl Med 2012;4:158rv11–158rv11. 10.1126/scitranslmed.3003528 PMC3618897 23115356

[55] Kurokawa J, Kodama M, Clancy CE, Furukawa T. Sex hormonal regulation of cardiac ion channels in drug-induced QT syndromes. Pharmacol Ther 2016;168:23–28. 27595633 10.1016/j.pharmthera.2016.09.004 PMC5140718

[56] Niemeijer MN, van den Berg ME, Deckers JW, Aarnoudse ALHJ, Hofman A, Franco OH, Uitterlinden AG, Rijnbeek PR, Eijgelsheim M, Stricker BH. ABCB1 gene variants, digoxin and risk of sudden cardiac death in a general population. Heart 2015;101:1973–1979. 26531821 10.1136/heartjnl-2014-307419

[57] Kim DW, Jang HY, Kim KW, Shin Y, Park SH. Design characteristics of studies reporting the performance of artificial intelligence algorithms for diagnostic analysis of medical images: results from recently published papers. Korean J Radiol 2019;20:405. 30799571 10.3348/kjr.2019.0025 PMC6389801

[58] Chang KC, Dutta S, Mirams GR, Beattie KA, Sheng J, Tran PN, Wu M, Wu WW, Colatsky T, Strauss DG, Li Z. Uncertainty quantification reveals the importance of data variability and experimental design considerations for in silico proarrhythmia risk assessment. Front Physiol 2017;8:917. 29209226 10.3389/fphys.2017.00917 PMC5702340

[59] Dey P, Ross JS, Ritchie JD, Desai NR, Bhavnani SP, Krumholz HM. Data sharing and cardiology. J Am Coll Cardiol 2017;70:3018–3025. 29241491 10.1016/j.jacc.2017.10.037

[60] Schiltz M. Science without publication paywalls: cOAlition S for the realisation of full and immediate open access. PLoS Med 2018;15:e1002663. 30178782 10.1371/journal.pmed.1002663 PMC6122176

[61] Britton OJ, Bueno-Orovio A, Van Ammel K, Lu HR, Towart R, Gallacher DJ, Rodriguez B. Experimentally calibrated population of models predicts and explains intersubject variability in cardiac cellular electrophysiology. Proc Natl Acad Sci USA 2013;110:E2098–E2105. 23690584 10.1073/pnas.1304382110 PMC3677477

[62] Sánchez C, Bueno-Orovio A, Wettwer E, Loose S, Simon J, Ravens U, Pueyo E, Rodriguez B. Inter-subject variability in human atrial action potential in sinus rhythm versus chronic atrial fibrillation. PLoS One 2014;9:e105897. 25157495 10.1371/journal.pone.0105897 PMC4144914

[63] Passini E, Britton OJ, Lu HR, Rohrbacher J, Hermans AN, Gallacher DJ, Greig RJH, Bueno-Orovio A, Rodriguez. Human in silico drug trials demonstrate higher accuracy than animal models in predicting clinical pro-arrhythmic cardiotoxicity. Front Physiol 2017;8: doi: 10.3389/fphys.2017.00668. 10.3389/fphys.2017.00668 PMC5601077 28955244

[64] Sánchez C, Bueno-Orovio A, Pueyo E, Rodríguez B. Atrial fibrillation dynamics and ionic block effects in six heterogeneous human 3D virtual atria with distinct repolarization dynamics. Front Bioeng Biotechnol 2017;5:29. 28534025 10.3389/fbioe.2017.00029 PMC5420585

[65] Liang L, Liu M, Martin C, Elefteriades JA, Sun W. A machine learning approach to investigate the relationship between shape features and numerically predicted risk of ascending aortic aneurysm. Biomech Model Mechanobiol 2017;16:1519–1533. 28386685 10.1007/s10237-017-0903-9 PMC5630492

[66] Sims CR, Delima LR, Calimaran A, Hester R, Pruett WA. Validating the physiologic model HumMod as a substitute for clinical trials involving acute normovolemic hemodilution. Anesth Analg 2018;126:93–101. 28863020 10.1213/ANE.0000000000002430

[67] Hernán MA, Robins JM. Using big data to emulate a target trial when a randomized trial is not available: table 1. Am J Epidemiol 2016;183:758–764. 26994063 10.1093/aje/kwv254 PMC4832051

[68] Colatsky T, Fermini B, Gintant G, Pierson JB, Sager P, Sekino Y, Strauss DG, Stockbridge N. The Comprehensive in Vitro Proarrhythmia Assay (CiPA) initiative—update on progress. J Pharmacol Toxicol Methods 2016;81:15–20. 27282641 10.1016/j.vascn.2016.06.002

[69] Cavero I, Holzgrefe H. CiPA: ongoing testing, future qualification procedures, and pending issues. J Pharmacol Toxicol Methods 2015;76:27–37. 26159293 10.1016/j.vascn.2015.06.004

[70] Pappalardo F, Russo G, Tshinanu FM, Viceconti M. In silico clinical trials: concepts and early adoptions. Brief Bioinform 2019:**20**:1699–1708. 10.1093/bib/bby043 29868882

[71] Viceconti M, Henney A, Morley-Fletcher E. In silico clinical trials: how computer simulation will transform the biomedical industry. Int J Clin Trials 2016;3:37.

[72] Haddad T, Himes A, Thompson L, Irony T, Nair R; MDIC Computer Modeling and Simulation Working Group Participants. Incorporation of stochastic engineering models as prior information in Bayesian medical device trials. J Biopharm Stat 2017;27:1089–1103. 28281931 10.1080/10543406.2017.1300907

[73] Faris O, Shuren J. An FDA viewpoint on unique considerations for medical-device clinical trials. N Engl J Med 2017;376:1350–1357. 28379790 10.1056/NEJMra1512592

[74] Kovatchev BP, Breton M, Man CD, Cobelli C. In silico preclinical trials: a proof of concept in closed-loop control of type 1 diabetes. J Diabetes Sci Technol 2009;3:44–55. 19444330 10.1177/193229680900300106 PMC2681269

[75] Zemzemi N, Bernabeu MO, Saiz J, Cooper J, Pathmanathan P, Mirams GR, Pitt-Francis J, Rodriguez B. Computational assessment of drug-induced effects on the electrocardiogram: from ion channel to body surface potentials. Br J Pharmacol 2013;168:718–733. 22946617 10.1111/j.1476-5381.2012.02200.x PMC3579290

[76] Rocatello G, El Faquir N, De Santis G, Iannaccone F, Bosmans J, De Backer O, Sondergaard L, Segers P, De Beule M, de Jaegere P, Mortier P. Patient-specific computer simulation to elucidate the role of contact pressure in the development of new conduction abnormalities after catheter-based implantation of a self-expanding aortic valve. Circ Cardiovasc Interv 2018;11:e005344. 29386188 10.1161/CIRCINTERVENTIONS.117.005344

[77] Andreu D, Ortiz-Pérez JT, Fernández-Armenta J, Guiu E, Acosta J, Prat-González S, De Caralt TM, Perea RJ, Garrido C, Mont L, Brugada J, Berruezo A. 3D delayed-enhanced magnetic resonance sequences improve conducting channel delineation prior to ventricular tachycardia ablation. Europace 2015;17:938–945. 25616406 10.1093/europace/euu310

[78] Cedilnik N, Duchateau J, Dubois R, Sacher F, Jaïs P, Cochet H,Sermesant M. Fast personalized electrophysiological models from computed tomography images for ventricular tachycardia ablation planning. Europace 2018;20:iii94–iii101. 30476056 10.1093/europace/euy228

[79] Kreimeyer K, Foster M, Pandey A, Arya N, Halford G, Jones SF, Forshee R, Walderhaug M, Botsis T. Natural language processing systems for capturing and standardizing unstructured clinical information: a systematic review. J Biomed Inform 2017;73:14–29. 28729030 10.1016/j.jbi.2017.07.012 PMC6864736

[80] Politou E, Alepis E, Patsakis C. Forgetting personal data and revoking consent under the GDPR: challenges and proposed solutions. J Cybersecurity 2018;4: doi: 10.1093/cybsec/tyy001.

[81] Darcy AM, Louie AK, Roberts LW. Machine learning and the profession of medicine. JAMA 2016;315:551. 26864406 10.1001/jama.2015.18421

[82] Esfandiari N, Babavalian MR, Moghadam A-ME, Tabar VK. Expert systems with applications knowledge discovery in medicine: current issue and future trend. Expert Syst Appl 2014;41:4434–4463.

[83] Nordling L. A fairer way forward for AI in health care. Nature 2019;573:S103–S105. 31554993 10.1038/d41586-019-02872-2

[84] Pathmanathan P, Cordeiro JM, Gray RA. Comprehensive uncertainty quantification and sensitivity analysis for cardiac action potential models. Front Physiol 2019;10:721. 31297060 10.3389/fphys.2019.00721 PMC6607060

[85] Miotto R, Li L, Kidd BA, Dudley JT. Deep patient: an unsupervised representation to predict the future of patients from the electronic health records. Sci Rep 2016;6:26094. 27185194 10.1038/srep26094 PMC4869115

[86] Land S, Gurev V, Arens S, Augustin CM, Baron L, Blake R, Bradley C, Castro S, Crozier A, Favino M, Fastl TE, Fritz T, Gao H, Gizzi A, Griffith BE, Hurtado DE, Krause R, Luo X, Nash MP, Pezzuto S, Plank G, Rossi S, Ruprecht D, Seemann G, Smith NP, Sundnes J, Rice JJ, Trayanova N, Wang D, Jenny Wang Z, Niederer SA. Verification of cardiac mechanics software: benchmark problems and solutions for testing active and passive material behaviour. Proc R Soc A Math Phys Eng Sci 2015;471:20150641. 10.1098/rspa.2015.0641 PMC4707707 26807042

[87] Niederer SA, Kerfoot E, Benson AP, Bernabeu MO, Bernus O, Bradley C, Cherry EM, Clayton R, Fenton FH, Garny A, Heidenreich E, Land S, Maleckar M, Pathmanathan P, Plank G, Rodríguez JF, Roy I, Sachse FB, Seemann G, Skavhaug O, Smith NP. Verification of cardiac tissue electrophysiology simulators using an N-version benchmark. Philos Trans R Soc A Math Phys Eng Sci 2011;369:4331–4351. 10.1098/rsta.2011.0139 PMC3263775 21969679

[88] Eden C, Johnson KW, Gottesman O, Bottinger EP, Abul-Husn NS. Medical student preparedness for an era of personalized medicine: findings from one US medical school. Per Med 2016;13:129–141. 27528879 10.2217/pme.15.58 PMC4982654

