# Supplementary material for: The ‘Digital Twin’ to enable the vision of precision cardiology
Source: Eur Heart J. 2020 Mar 4;41(48):4556–64. doi: 10.1093/eurheartj/ehaa159 (PMC7774470; doi:10.1093/eurheartj/ehaa159)
Supplement: ehaa159_Supplementary_Data [file ehaa159_supplementary_data.pdf]

The "digital twin" to enable the vision of precision cardiology

Supplemental material: Mechanistic and statistical model synergy for exploiting and integrating clinical data

There is an increasing availability of clinical data from diverse sources and modalities that can be harnessed through the digital twin paradigm. The healthcare sector has often been indicated as one of the industries that could benefit the most from big data technology[1] that is demonstrating the potential impact in cardiovascular care[2–5]. Examples include rapid analysis of image data[6] or the discovery of new patterns or signatures of disease[7,8]. Statistical models to exploit the big data bring the promise of increased efficiency, accuracy, and reproducibility to clinical care. However, while preliminary results exist, many of these models have not yet been translated into the clinical workflow, and this section illustrates how an increased synergy with mechanistic models can accelerate this translation.

Modelling approaches for image analysis

A natural interplay between mechanistic and statistical models occurs through the images that are used to inform models, and both modelling approaches are driven by progress in imaging technology[9]. Images can be used to generate patient-specific mechanistic models of cardiac function as well as to extract knowledge using statistical models. To date, these tasks have primarily been handled separately but harnessing the interplay between these modelling types offers opportunities to advance both.

Mechanistic models can benefit from the ability of statistical models to automatically segment and extract landmarks from images. The generation of patient-specific models from imaging data traditionally required considerable effort. Image registration and/or segmentation techniques have now become the core engine for this personalisation[10,11], and these processes can now benefit from using fully automated convolutional neural network-based approaches[12–14].

There is an increasing recognition that mechanistic models can also be used to improve the accuracy and reliability of statistical models in image-based analysis, which have experienced an explosion in

the medical field over the last decade[15,16]. However, the accuracy of these models depends on accurate and reliable annotation of the training data. The high inter-observer and intra-observer variability, seen e.g. in common ultrasound measurements[17], makes this reliability questionable. Either separate labelling studies must be commissioned to ensure data reliability or extensive data cleaning processes must be undertaken, both time-consuming and expensive due to the large amount of training data required. Mechanistic models can be used here as tools to simulate new sets of training data[18]. Large sets of synthetic patients can be generated with a known ground truth by varying parameters in mechanistic models, and data can then be generated using emulators of the image acquisition process[19,20].

The interplay between mechanistic and statistical models can also expand to the inference of cardiac function from images. An existing example is the use of a mechanistic model of cardiac electrical propagation to train a statistical model and then infer response to cardiac resynchronisation therapy (CRT) from body surface potential maps, achieving good predictive power when tested in patient data[21].

The area of image analysis is where industrial translation has generated early success stories, enabled by different degrees of integration between statistical and mechanistic models. For example, CardioAI (Arterys Inc, USA) has implemented deep learning algorithms for segmentation[22], magnetic resonance view projection[23], and data generation[24]. EchoMD AutoEF (Bay Labs Inc, USA) assists the acquisition process with scan quality assessment in echocardiography, and EchoGo (Ultromics, UK) provides diagnostic assistance in stress-echo scans[25]. The benefits of these products include reducing operation time and sharing expertise and data for better model training.

Models to study molecular profiling data

Over the past decades, multiple -omics profiling technologies have emerged and been used in a wide variety of medical fields. In cardiology, numerous studies have demonstrated the complexity of cardiovascular diseases as intricate interactions of many genes, non-coding regions and regulatory proteins[26,27]. These technologies are also becoming more affordable, increasing their presence and enabling the creation of bigger databases that will provide new insights into cardiovascular disease pathophysiology[28].

In a clinical environment, polygenic risk scores are currently used to discover at-risk patients by providing a forecast of cardiovascular events like myocardial infarction, heart failure and stroke, and to help customise therapy selection. Recent studies have shown different promising applications for molecular profiling, like their combination with imaging to compile a stratified profile of patient population[29], or using these approaches to help identifying new target molecules for drug discovery and repositioning[30]. And high-throughput protein profiling methods are increasing specificity of protein read-outs in multiplexing assays[31,32].

One challenge in these studies is the very large and heterogeneous data, with many variations to be stored, analysed and used. There is growing evidence of the important role that statistical models could have in cardiovascular disease risk estimation by computing personalised genomic risk scores[33]. Several studies have already improved performance by applying machine learning to conventional cardiovascular disease risk factors on large populations[34,35]. An example is the ability to assess, in abdominal aortic aneurysm, the effectiveness of adjusting lifestyles given personal genome baselines by integrating personal genomes and electronic health record data, demonstrating its utility as a personalised health management[36].

Mechanistic models are also being used to refine our understanding of genomic variants in cardiac disease. Examples include elucidating the role of early somatic mosaicism in life-threatening arrhythmias in long-QT syndrome infants[37], characterising novel autosomal dominant heterozygous mutations in catecholaminergic polymorphic ventricular tachycardia[38], or explaining juxtaposed effects of gain-of-function mutations linked to QT prolongation and sudden cardiac death when expected to cause QT shortening[39]. The integration of statistical and mechanistic models holds significant potential for identifying novel genotypes and phenotypes in heterogeneous cardiovascular diseases[40]. In a near future, such datasets could be generated from patient samples and become a routine part of cardiovascular care and diagnosis, which could then feed mechanistic models to provide better insights into human biology.

Models for home monitoring and wearable sensors

With commercial technologies developing at a fast pace, even highly reliable and accurate acquisition devices can now be deployed in ambulatory or domestic care scenarios. Smart watches and wearable sensors are opening a new dimension for continuous monitoring and, subsequently, for diagnosing and detecting critical health events.

As an example, ambulatory ECG measurements have the potential of early detection of atrial fibrillation, leading to refined health care resource use and a more timely initiation of anticoagulant therapy[56], with logic behind the ECG signal analysis based on the mechanistic understanding of rhythm variability. The opportunity for improvements resides in applying machine learning methods to identify additional signatures in long duration ECG recordings[57]. Other early examples have demonstrated the ability of statistical models to handle and analyse vast datasets from wearable devices in a clinically meaningful way. Patterns extracted from photoplethysmography signals were used to detect atrial fibrillation in an ambulatory setting, showcasing the potential of these methods in early detection[58]. Furthermore, mechanistic-driven processing of the photoplethysmography signal has been applied to identify heart rate variability[59].

Models for population studies

The inherent limitations of shaping treatment guidelines based on large population studies leads to decisions that are based on an "average" patient within a large group, thereby missing the opportunity of personalisation. In this setting, the digital twin may present an opportunity to define targeted patient-specific guidelines based on standardised predictive models taking in account the "individual-specific" factors for treatment[41].

Furthermore, the increasing availability of large cardiological databases in electronic health records, and the integration with imaging, omics and wearable/home sources, is creating opportunity to improve disease diagnosis and prognosis. Building on these records, information gained on a patient population can be used to individualise care[42,43], or to build risk prediction models using records from different countries[44–46]. The clinical adoption of these tools relies on their validity, and thus on the availability of

multiple databases with good data recording control to prevent bias and missing data. However, combining databases is challenging in practice. The majority share basic categories of information such as age or undergone procedures but may lack specific examination findings such as ejection fraction or haemoglobin level.

Statistical and mechanistic models can tackle these problems. Sensitivity analyses can determine the most important factors, and data imputation may help addressing incomplete records. Models can simulate the set of missing data[47], and can then be used to assist the personalisation of treatment for individual patients[41,48,49]. For example, collaborative filtering techniques can integrate data from multiple sources to provide an estimate in cases where data points are missing[50]. Such approaches have already shown high predictive accuracy for both sudden cardiac death and recurrent myocardial infarction[51].

Other machine learning techniques such as principal component analysis or kernel learning can also determine the most relevant dimensions of data sources with hundreds or thousands of dimensions[52,53]. This can be used to identify the most relevant parameters for mechanistic models. Similarly, sensitivity and uncertainty analyses have been employed with mechanistic models to identify important parameters in simulations[54,55], thus guiding the choice of the most relevant metrics for population studies.

References

1. Murdoch TB, Detsky AS. The Inevitable Application of Big Data to Health Care. *JAMA* American Medical Association; 2013;**309**:1351.

2. Wang W, Krishnan E. Big data and clinicians: A review on the state of the science. J. Med. Internet Res. JMIR Publications Inc.; 2014. p. e1.

3. Jee K, Kim G-H. Potentiality of big data in the medical sector: focus on how to reshape the healthcare system. *Healthc Inform Res* Korean Society of Medical Informatics; 2013;**19**:79–85.

4. Johnson KW, Torres Soto J, Glicksberg BS, Shameer K, Miotto R, Ali M, Ashley E, Dudley JT. Artificial Intelligence in Cardiology. *J Am Coll Cardiol* Journal of the American College of Cardiology; 2018;**71**:2668–2679.

5. Rumsfeld JS, Joynt KE, Maddox TM. Big data analytics to improve cardiovascular care: promise and challenges. *Nat Rev Cardiol* 2016;**13**:350–359.

6. Dey D, Slomka PJ, Leeson P, Comaniciu D, Shrestha S, Sengupta PP, Marwick TH. Artificial

Intelligence in Cardiovascular Imaging: JACC State-of-the-Art Review. *J Am Coll Cardiol* Elsevier; 2019;**73**:1317–1335.

7. Shen D, Wu G, Suk H-I. Deep Learning in Medical Image Analysis. *Annu Rev Biomed Eng* 2017;**19**:221–248.

8. Gibson E, Hu Y, Huisman HJ, Barratt DC. Designing image segmentation studies: Statistical power, sample size and reference standard quality. *Med Image Anal* 2017;**42**:44–59.

9. Lamata P, Casero R, Carapella V, Niederer SASA, Bishop MJMJ, Schneider JEJE, Kohl P, Grau V. Images as drivers of progress in cardiac computational modelling. *Prog Biophys Mol Biol* 2014;**115**:198–212.

10. Lamata P, Niederer S, Nordsletten D, Barber DCDC, Roy I, Hose DR, Smith N. An accurate, fast and robust method to generate patient-specific cubic Hermite meshes. *Med Image Anal* Elsevier; 2011;**15**:801–813.

11. Crozier A, Augustin CM, Neic A, Prassl AJ, Holler M, Fastl TE, Hennemuth A, Bredies K, Kuehne T, Bishop MJ, Niederer SA, Plank G. Image-Based Personalization of Cardiac Anatomy for Coupled Electromechanical Modeling. *Ann Biomed Eng* Springer US; 2016;**44**:58–70.

12. Bai W, Sinclair M, Tarroni G, Oktay O, Rajchl M, Vaillant G, Lee AM, Aung N, Lukaschuk E, Sanghvi MM, Zemrak F, Fung K, Paiva JM, Carapella V, Kim YJ, Suzuki H, Kainz B, Matthews PM, Petersen SE, Piechnik SK, Neubauer S, Glocker B, Rueckert D. Automated cardiovascular magnetic resonance image analysis with fully convolutional networks. *J Cardiovasc Magn Reson* 2018;**20**:65.

13. Vos BD de, Berendsen FF, Viergever MA, Staring M, Išgum I. End-to-End Unsupervised Deformable Image Registration with a Convolutional Neural Network. Springer, Cham; 2017. p. 204–212.

14. Corral Acero J, Zacur E, Xu H, Ariga R, Bueno-Orovio A, Lamata P, Grau V. SMOD - Data Augmentation Based on Statistical Models of Deformation to Enhance Segmentation in 2D Cine Cardiac MRI. Springer, Cham; 2019. p. 361–369.

15. Litjens G, Kooi T, Bejnordi BE, Arindra A, Setio A, Ciompi F, Ghafoorian M, Laak JAWM Van Der, Ginneken B Van, Sánchez CI. A Survey on Deep Learning in Medical Image Analysis.

16. Sahiner B, Pezeshk A, Hadjiiski LM, Wang X, Drukker K, Cha KH, Summers RM, Giger ML. Deep learning in medical imaging and radiation therapy. *Med Phys* John Wiley & Sons, Ltd; 2019;**46**:e1–e36.

17. Thorstensen A, Dalen H, Amundsen BH, Aase SA, Stoylen A. Reproducibility in echocardiographic assessment of the left ventricular global and regional function, the HUNT study. *Eur J Echocardiogr* Narnia; 2010;**11**:149–156.

18. Achille P Di, Harouni A, Khamzin S, Solovyova O, Rice JJ, Gurev V. Gaussian Process Regressions for Inverse Problems and Parameter Searches in Models of Ventricular Mechanics. *Front Physiol* 2018;**9**:1002.

19. Lawson BA, Burrage K, Burrage P, Drovandi CC, Bueno-Orovio A. Slow Recovery of Excitability Increases Ventricular Fibrillation Risk as Identified by Emulation. *Front Physiol* Frontiers; 2018;**9**:1114.

20. Chang ETY, Strong M, Clayton RH. Bayesian Sensitivity Analysis of a Cardiac Cell Model Using a Gaussian Process Emulator. Burrage K, ed. *PLoS One* 2015;**10**:e0130252.

21. Giffard-Roisin S, Delingette H, Jackson T, Webb J, Fovargue L, Lee J, Rinaldi CA, Razavi R,

Ayache N, Sermesant M. Transfer Learning From Simulations on a Reference Anatomy for ECGI in Personalized Cardiac Resynchronization Therapy. *IEEE Trans Biomed Eng* 2019;**66**:343–353.

22. Lieman-Sifry J, Le M, Lau F, Sall S, Golden D. Fastventricle: Cardiac segmentation with ENet. *Lecture Notes in Computer Science (including subseries Lecture Notes in Artificial Intelligence and Lecture Notes in Bioinformatics)* 2017. p. 127–138.

23. Le M, Lieman-Sifry J, Lau F, Sall S, Hsiao A, Golden D. Computationally efficient cardiac views projection using 3D convolutional neural networks. *Lecture Notes in Computer Science (including subseries Lecture Notes in Artificial Intelligence and Lecture Notes in Bioinformatics)* 2017. p. 109–116.

24. Lau F, Hendriks T, Lieman-Sifry J, Norman B, Sall S, Golden D. ScarGAN: Chained Generative Adversarial Networks to Simulate Pathological Tissue on Cardiovascular MR Scans. 2018;

25. Alsharqi M, Upton R, Mumith A, Leeson P. Artificial intelligence: a new clinical support tool for stress echocardiography. *Expert Rev Med Devices* Taylor & Francis; 2018;**15**:513–515.

26. Deloukas P, Kanoni S, Genotyping AZ, Absher : D, Consortium SC-B. Large-scale association analysis identifies new risk loci for coronary artery disease. *AUTHOR Contrib Writ* 2013;**45**:25–33.

27. Shameer K, Denny JC, Ding K, Jouni H, Crosslin DR, Andrade M De, Chute CG, Peissig P, Pacheco JA, Li R, Bastarache L, Kho AN, Ritchie MD, Masys DR, Chisholm RL, Larson EB, Mccarty CA, Roden DM, Jarvik GP, Kullo IJ. A Genome-and Phenome-Wide Association Study to Identify Genetic Variants Influencing Platelet Count and Volume and their Pleiotropic Effects. *Hum Genet* 2014;**133**.

28. Shameer K, Johnson KW, Glicksberg BS, Dudley JT, Sengupta PP. Machine learning in cardiovascular medicine: are we there yet? *Heart* 2018;**104**:1156–1164.

29. Kini AS, Vengrenyuk Y, Shameer K, Maehara A, Purushothaman M, Yoshimura T, Matsumura M, Aquino M, Haider N, Johnson KW, Readhead B, Kidd BA, Feig JE, Krishnan P, Sweeny J, Milind M, Moreno P, Mehran R, Kovacic JC, Baber U, Dudley JT, Narula J, Sharma S. Intracoronary Imaging, Cholesterol Efflux, and Transcriptomes After Intensive Statin Treatment: The YELLOW II Study. *J Am Coll Cardiol* Elsevier; 2017;**69**:628–640.

30. Shameer K, Glicksberg BS, Hodos R, Johnson KW, Badgeley MA, Readhead B, Tomlinson MS, O’connor T, Miotto R, Kidd BA, Chen R, Ma’ayan A, Dudley JT. Systematic analyses of drugs and disease indications in RepurposeDB reveal pharmacological, biological and epidemiological factors influencing drug repositioning. *Brief Bioinform* 2018;**19**:656–678.

31. Joshi A, Mayr M. In Aptamers They Trust. *Circulation* 2018;**138**:2482–2485.

32. Lind L, Ärnlöv J, Lindahl B, Siegbahn A, Sundström J, Ingelsson E. Use of a proximity extension assay proteomics chip to discover new biomarkers for human atherosclerosis. *Atherosclerosis* 2015;**242**:205–210.

33. Kullo IJ, Jouni H, Austin EE, Brown S-A, Kruisselbrink TM, Isseh IN, Haddad RA, Marroush TS, Shameer K, Olson JE, Broeckel U, Green RC, Schaid DJ, Montori VM, Bailey KR. Incorporating a Genetic Risk Score into Coronary Heart Disease Risk Estimates: Effect on LDL Cholesterol Levels (the MIGENES Clinical Trial). *Circ J* 2016;

34. Weng SF, Reps J, Kai J, Garibaldi JM, Qureshi N. Can machine-learning improve cardiovascular risk prediction using routine clinical data? Liu B, ed. *PLoS One* Public Library of Science; 2017;**12**:e0174944.

35. Zhao J, Feng Q, Wu P, Lupu RA, Wilke RA, Wells QS, Denny JC, Wei W-Q. Learning from Longitudinal Data in Electronic Health Record and Genetic Data to Improve Cardiovascular Event Prediction. *Sci Rep* Nature Publishing Group; 2019;**9**:717.

36. Li J, Pan C, Zhang S, Spin JM, Deng A, Leung LLK, Dalman RL, Tsao PS, Snyder M. Decoding the Genomics of Abdominal Aortic Aneurysm. *Cell* Cell Press; 2018;**174**:1361--1372.e10.

37. Priest JR, Gawad C, Kahlig KM, Yu JK, O'Hara T, Boyle PM, Rajamani S, Clark MJ, Garcia STK, Ceresnak S, Harris J, Boyle S, Dewey FE, Malloy-Walton L, Dunn K, Grove M, Perez M V., Neff NF, Chen R, Maeda K, Dubin A, Belardinelli L, West J, Antolik C, Macaya D, Quertermous T, Trayanova NA, Quake SR, Ashley EA. Early somatic mosaicism is a rare cause of long-QT syndrome. *Proc Natl Acad Sci* 2016;**113**:11555–11560.

38. Gray B, Bagnall RD, Lam L, Ingles J, Turner C, Haan E, Davis A, Yang P-C, Clancy CE, Sy RW, Semsarian C. A novel heterozygous mutation in cardiac calsequestrin causes autosomal dominant catecholaminergic polymorphic ventricular tachycardia. *Hear Rhythm* 2016;**13**:1652–1660.

39. Zhou X, Bueno-Orovio A, Schilling RJ, Kirkby C, Denning C, Rajamohan D, Burrage K, Tinker A, Rodriguez B, Harmer SC. Investigating the Complex Arrhythmic Phenotype Caused by the Gain-of-Function Mutation KCNQ1-G229D. *Front Physiol* 2019;**10**:259.

40. Krittanawong C, Zhang H, Wang Z, Aydar M, Kitai T. Artificial Intelligence in Precision Cardiovascular Medicine. *J Am Coll Cardiol* Elsevier; 2017;**69**:2657–2664.

41. Gray RA, Pathmanathan P. Patient-specific cardiovascular computational modeling: Diversity of personalization and challenges. *J Cardiovasc Transl Res* Journal of Cardiovascular Translational Research; 2018;**11**:80–88.

42. Ng K, Steinhubl SR, Defilippi C, Dey S, Stewart WF. Early Detection of Heart Failure Using Electronic Health Records: Practical Implications for Time before Diagnosis, Data Diversity, Data Quantity, and Data Density. *Circ Cardiovasc Qual Outcomes* 2016;**9**:649–658.

43. Panahiazar M, Taslimitehrani V, Pereira NL, Pathak J. Using EHRs for Heart Failure Therapy Recommendation Using Multidimensional Patient Similarity Analytics. *Stud Health Technol Inform* 2016;**210**:6072–6078.

44. Zarrinkoub R, Wettermark B, Wändell P, Mejhert M, Szulkin R, Ljunggren G, Kahan T. The epidemiology of heart failure, based on data for 2.1 million inhabitants in Sweden. *Eur J Heart Fail* 2013;**15**:995–1002.

45. Gho JMIH, Schmidt AF, Pasea L, Koudstaal S, Pujades-Rodriguez M, Denaxas S, Shah AD, Patel RS, Gale CP, Hoes AW, Cleland JG, Hemingway H, Asselbergs FW. An electronic health records cohort study on heart failure following myocardial infarction in England: Incidence and predictors. *BMJ Open* 2018;**8**:1–10.

46. Ohlmeier C, Mikolajczyk R, Frick J, Prütz F, Haverkamp W, Garbe E. Incidence, prevalence and 1-year all-cause mortality of heart failure in Germany: a study based on electronic healthcare data of more than six million persons. *Clin Res Cardiol* 2015;**104**:688–696.

47. Beaulieu-Jones BK, Moore JH. Missing Data Imputation in the Electronic Health Record Using Deeply Learned Autoencoders. *Pacific Symposium on Biocomputing* 2017. p. 208.

48. Kayvanpour E, Mansi T, Sedaghat-Hamedani F, Amr A, Neumann D, Georgescu B, Seegerer P, Kamen A, Haas J, Frese KS, Irawati M, Wirsz E, King V, Buss S, Mereles D, Zitron E, Keller A, Katus HA, Comaniciu D, Meder B. Towards personalized cardiology: Multi-scale modeling of the failing heart. *PLoS One* 2015;**10**:1–18.

49. Panahiazar M, Taslimitehrani V, Pereira N, Pathak J. Using EHRs and Machine Learning for Heart Failure Survival Analysis. *Stud Health Technol Inform* 2015;**216**:40–44.

50. Alhamid MF, Rawashdeh M, Osman H Al, Saddik A El. Leveraging biosignal and collaborative filtering for context-aware recommendation. *MIIRH '13 Proceedings of the 1st ACM international workshop on Multimedia indexing and information retrieval for healthcare* 2013. p. 41–48.

51. Shahzain H, Syed Z. From netflix to heart attacks: Collaborative filtering in medical datasets. *IHI'10 - Proceedings of the 1st ACM International Health Informatics Symposium* 2010. p. 128–134.

52. Saraçoglu R. Hidden Markov model-based classification of heart valve disease with PCA for dimension reduction. *Eng Appl Artif Intell* 2012;**25**:1523–1528.

53. Xia Y. A multiple-index model and dimension reduction. *J Am Stat Assoc* 2008;**103**:1631–1640.

54. Wencker D, Armstrong RC, Kitsis RN, Wencker D, Chandra M, Nguyen K, Miao W, Garantziotis S, Factor SM, Shirani J, Armstrong RC, Kitsis RN. A mechanistic role for cardiac myocyte apoptosis in heart failure Find the latest version : A mechanistic role for cardiac. *J Clin Invest* 2003;**111**:1497–1504.

55. Pathmanathan P, Gray RA. Ensuring reliability of safety-critical clinical applications of computational cardiac models. *Front Physiol* Frontiers; 2013;**4**:358.

56. Steinhubl SR, Waalen J, Edwards AM, Ariniello LM, Mehta RR, Ebner GS, Carter C, Baca-Motes K, Felicione E, Sarich T, Topol EJ. Effect of a Home-Based Wearable Continuous ECG Monitoring Patch on Detection of Undiagnosed Atrial Fibrillation. *JAMA* American Medical Association; 2018;**320**:146.

57. Mincholé A, Rodriguez B. Artificial intelligence for the electrocardiogram. *Nat Med* Nature Publishing Group; 2019;**25**:22–23.

58. Tison GH, Sanchez JM, Ballinger B, Singh A, Olgin JE, Pletcher MJ, Vittinghoff E, Lee ES, Fan SM, Gladstone RA, Mikell C, Sohoni N, Hsieh J, Marcus GM. Passive Detection of Atrial Fibrillation Using a Commercially Available Smartwatch. *JAMA Cardiol* American Medical Association; 2018;**3**:409.

59. Gil E, Orini M, Bailón R, Vergara JM, Mainardi L, Laguna P. Photoplethysmography pulse rate variability as a surrogate measurement of heart rate variability during non-stationary conditions. *Physiol Meas* IOP Publishing; 2010;**31**:1271–1290.
